# Circular RNA hsa_circ_0081343 promotes trophoblast cell migration and invasion and inhibits trophoblast apoptosis by regulating miR-210-5p/DLX3 axis

**DOI:** 10.1186/s12958-021-00795-0

**Published:** 2021-08-09

**Authors:** Hui Wang, Caiqun Luo, Xiaoxia Wu, Jianming Zhang, Zhiyong Xu, Yang Liu, Bohong Li, Jing Li, Jiansheng Xie

**Affiliations:** 1 Department of Obstetrics and Gynecology, Shenzhen Maternity and Child Healthcare Hospital, Southern Medical University, Shenzhen, 518000 Guangdong China; 2 Institute of Maternal and Child Medicine, Shenzhen Maternity and Child Healthcare Hospital, Southern Medical University, Shenzhen, 518000 Guangdong China; 3 Medical genetic center, Shenzhen Maternity and Child Healthcare Hospital, Southern Medical University, Shenzhen, 518000 Guangdong China; 4 Department of Obstetrics and Gynecology, Nanfang Hospital, Southern Medical University, Guangzhou, 510515 Guangdong China

**Keywords:** Circular RNA, hsa_circ_0081343, Fetal growth restriction, HTR-8, Competing endogenous RNA, miR-210-5p

## Abstract

**Background:**

Various circular RNAs (circRNAs) are dysregulated in the placenta of fetal growth restriction (FGR) fetuses, but their roles and regulatory mechanisms have not been fully elucidated. Herein, we aimed to elucidate the role of hsa_circ_0081343 in regulating the migration, invasion, and apoptosis of human extravillous trophoblast HTR-8 cells.

**Methods:**

CircRNA and miRNA levels were examined by quantitative reverse transcription PCR (qRT-PCR). Overexpression plasmid constructs and siRNAs were used to overexpress and knockdown hsa_circ_0081343, respectively. Transwell assays and flow cytometry analyses were performed to evaluate the effects of hsa_circ_0081343 or miR-210-5p on migration, invasion, and apoptosis. Protein levels were analyzed by western blotting. Dual luciferase activity and anti-AGO2 RNA immunoprecipitation (RIP) assays were performed to identify the relationship between miR-210-5p and hsa_circ_0081343.

**Results:**

Hsa_circ_0081343 expression was significantly downregulated in 37 FGR placental tissues compared to healthy placental control tissues. Hsa_circ_0081343 overexpression may inhibit apoptosis by downregulating the expression of cleaved caspase 3 and caspase 9 and alleviating the migration and invasion of HTR-8 cells by inducing the expression of MMP2 and MMP9. The dual luciferase activity and anti-AGO2 RIP assay results showed that hsa_circ_0081343 binds to miR-210-5p. miR-210-5p overexpression eliminated the effect of hsa_circ_0081343 overexpression in HTR-8 cells. Finally, DLX3 was identified as a direct target of miR-210-5p.

**Conclusions:**

hsa_circ_0081343 expression levels are significantly downregulated in FGR placental tissues. Hsa_circ_0081343 regulates the migration, invasion, and apoptosis of HTR-8 cells via the hsa-miR-210-5p/DLX3 axis.

## Background

Fetal growth restriction (FGR) refers to a condition in which the fetus does not maintain its intrauterine potential for growth and development. This condition further results in the fetal birth weight being less than 2500 g after 37 weeks of gestation, which is below the 10^th^ percentile of normal weight or less than two standard deviations below the mean weight for the same gestational age [1, 2]. It is a relatively common obstetric condition with a prevalence of 5–10% in all pregnancies and a contribution to 30% of stillbirths [3–5]. FGR is a multifactorial disorder, and the associated placental dysfunction has been linked to the deceleration of fetal growth. This dysfunction can result in a reduction in oxygen and nutrient supply from the mother to the fetus [5]. However, the precise molecular mechanisms underlying placental development and function remain unclear.

Circular RNAs (circRNAs) are a class of single-stranded endogenous molecules [6]. Their roles in the pathological processes of many diseases, especially cancers, have been widely discussed [6, 7]. CircRNAs are widely expressed in mammalian cells and are involved in the regulation of gene expression at the transcriptional or post-transcriptional level [8, 9]. Recent studies have found that circRNAs can bind to microRNAs and function as competing endogenous RNAs (ceRNA), bind to RNA-binding proteins to regulate gene transcription, or undergo translation as a template [10, 11]. Although circRNAs are regarded as important biological molecules and are associated with disease pathogenesis, the role of circRNAs in FGR is not well documented.

In our previous study, we identified differentially expressed circRNAs in FGR placenta using circRNA microarrays [12]. We found that hsa_circ_0000848, which is one of the top three differentially expressed circRNAs, is involved in regulating migration, invasion, and apoptosis of trophoblast cells [12], suggesting that it may play a role in the etiology and pathogenesis of FGR. However, the pathology of FGR is complex and other differentially expressed circRNAs may also be involved in this process. Therefore, in the present study, we aimed to further confirm the expression profile, functional role, and molecular mechanism of another differentially expressed circRNA, hsa_circ_0081343, which is one of the top six differentially expressed circRNAs. Similar to our previous study, HTR-8/SVneo cells were chosen as the in vitro cell model in the present study. The placenta is a multifunctional organ that is essential for fetal development and survival. Trophoblast cells are specialized cells in the placenta that mediate interactions between the fetus and mother at the fetomaternal interface. HTR-8/SVneo is a mature trophoblast cell model that maintains the basic characteristics of the original cells. Hence, it is currently used as a representative cell model of conditions such as FGR or preeclampsia.

## Materials and methods

### Tissue samples

The 37 pregnant women with FGR and 37 healthy pregnant women enrolled in this study were the same as those in our previous study [12]. Their detailed characteristics were reported in our previous study. Placental tissues were collected in RNA later (Sangon Biotech, Shanghai, China) after delivery and frozen in liquid nitrogen. This study was approved by the Ethics Committee of the Shenzhen Maternity and Child Health Hospital. Written informed consent was obtained from all patients.

### RNA isolation and quantitative reverse transcription PCR (qRT-PCR) assay

Total RNA from tissue samples or cells was extracted using TRIzol reagent (Invitrogen, Carlsbad, CA, USA) and quantified using the NanoDrop ND-1000. Reverse transcription (RT) was performed to obtain cDNA using the ImProm-IITM Reverse Transcription System (Promega, Madison, WI, USA). Random primers were used as the RT primers for detecting circRNA. The RT primer for detecting miRNAs was a special stem-loop primer based on the principle of the stem-loop method. Quantitative PCR analysis was performed using the SYBR GREEN qPCR Super Mix (Promega). GAPDH was used as the internal control for circRNA and mRNA. U6 was used as the internal control for miRNA. All assays were performed with three independent experiments. The data were calculated using the 2^−ΔΔCt^ method to represent the relative expression levels of the RNAs. The primers used for qRT-PCR are shown below. The forward and reverse primer sequences for hsa_circ_0081343 were AACGAGAACAAGTTTGCTGTG and AGTCGATGCCAGTCATTCTC, respectively. The forward and reverse primer sequences for GAPDH were GGGAAACTGTGGCGTGAT and GAGTGGGTGTCGCTGTTGA, respectively. The forward primers for miR-210-5p, miR-545-3p, and miR-597-3p were ACACTCCAGCTGGGAGCCCCTGCCCACCGCACAC, ACACTCCAGCTGGGTCAGCAAACATTTATTGTG, and ACACTCCAGCTGGGTGGTTCTCTTGTGGCTCA, respectively. The universal reverse primer for all miRNAs was CTCAACTGGTGTCGTGGA. The forward and reverse primer sequences for U6 (RNA, U6 small nuclear 1) were CTCGCTTCGGCAGCACA and AACGCTTCACGAATTTGCGT, respectively.

### Cell culture and transfection

HTR-8/SVneo cells were purchased from the American Type Culture Collection (Manassas, VA, USA) and cultured in RPMI 1640 (Gibco, Carlsbad, CA, USA) supplemented with 10% fetal bovine serum (Gibco), 1% penicillin/streptomycin, and 1% L-glutamine at 37 °C in a humidified incubator with 5% CO_2_. The full-length hsa_circ_0081343 (position: chr7:98,985,662–98,985,884; spliced length: 223 nt) was cloned into the pLCD5H-ciR plasmid (Guangzhou Geneseed Biotech Co., Ltd, China) by in vitro DNA synthesis to construct the hsa_circ_0081343 overexpression vector (ov-circ_0081343). Empty pLCD5H-ciR plasmid was used as a negative control (NC). Two small interfering RNAs (siRNAs) targeting hsa_circ_0081343 and named siRNA-1 (sense sequence: GGAGAAUGACUGGCAUCGATT) and siRNA-2 (sense sequence: GACUGGCAUCGACUGGGCCTT) were designed to include splice junctions to avoid degrading linear mRNA, which is then processed into circRNA. The sense sequence of the negative control siRNA (si-NC) was UUCUCCGAACGUGUCACGUTT. The negative control miRNA (miR-NC, UCACAACCUCCUAGAAAGAGUAGA), miR-210-5p mimics (AGCCCCUGCCCACCGCACACUG), miR-210-5p inhibitor (CAGUGUGCGGUGGGCAGGGGCU), and miR-NC inhibitor (UCUACUCUUUCUAGGAGGUUGUGA) were synthesized by GenePharma Co. (Shanghai, China).

The HTR-8 cells were seeded in six-well plates and transfected using Lipofectamine 2000 reagent (Invitrogen) according to the manufacturer's protocol. To investigate the effect of hsa_circ_0081343, the HTR-8 cells were divided into four groups: si-NC group transfected with si-NC, si-circ_0081343 group transfected with siRNA-2, NC group transfected with empty pLCD5H-ciR plasmid, and ov-circ_0081343 group transfected with ov-circ_0081343. To investigate whether miR-210-5p overexpression alleviates the effect of hsa_circ_0081343 overexpression, the HTR-8 cells were divided into three groups and transfected with one of the following schemes: ov-circ_0081343 + miR-NC, ov-circ_0081343 + miR-210-5p, or NC + miR-NC.

### Transwell assay and flow cytometry analysis

After 24 h of transfection, Transwell assays were performed to examine the migration and invasion capabilities of the indicated groups using the same method as described in our previous study [12]. The migrated or invaded cells at the bottom of the filter membrane were photographed using a light microscope (Nikon, Tokyo, Japan) at 200 × magnification. The cell number was counted in each photograph of five randomly selected fields, and the average was used as the number of cells that migrated or invaded per field. Each experiment was repeated three times.

After 24 h of transfection, the cells were analyzed for apoptosis using flow cytometry. Cell staining was performed using an Annexin V-FITC/PI Apoptosis Detection Kit (KeyGEN Biotech, Nanjing, China), and apoptosis levels were analyzed by flow cytometry using the same method as described previously [12].

### Western blot analysis

The concentrations of total proteins extracted using RIPA strong buffer (Beyotime, Shanghai, China) were quantified using the Bio-Rad Protein Assay Kit (Bio-Rad Laboratories, Hercules, CA, USA). Western blot analysis was performed using the method described in our previous study [12]. The primary antibodies against MMP2, MMP9, caspase 3, and caspase 9 were the same as those used in our previous study [12]. Anti-DLX3 antibody (1:800, ab64953) was also used.

### Dual luciferase activity assay

The fragments of wild-type linear hsa_circ_0081343 and the DLX3 3′ untranslated region (UTR) were amplified and cloned into the dual-luciferase miRNA target expression vector GP-mirGLO (Promega) and named as wild type circ_0081343 and wild type 3′ UTR, respectively. The binding sequence of miR-210-5p on the wild-type circ_0081343 and wild-type 3′ UTR plasmids were mutated by site-directed mutagenesis using one-step overlap extension PCR, and named as mutant circ_0081343 and mutant 3′ UTR, respectively. HTR-8 cells were plated on 24-well plates and co-transfected with 100 ng of the indicated recombinant plasmids and 50 nM of miR-210-5p mimic or miR-NC. After 48 h of transfection, firefly and *Renilla* luciferase activities were measured using the Dual-Luciferase Reporter Assay System (Promega) according to the manufacturer's instructions. Three independent experiments were conducted.

### Anti-AGO2 RNA immunoprecipitation (RIP) assay

RIP was performed according to the instructions included with the Magna RIP RNA-Binding Protein Immunoprecipitation Kit (Millipore, Bedford, MA, USA). Briefly, approximately 1 × 10^7^ cells were harvested after transfection with the miR-210-5p mimic or miR-NC. The cell pellets were lysed in polysome lysis buffer supplemented with protease inhibitor cocktail and RNase inhibitor. Partial cell lysate (20 μL), termed input, was collected for use as a positive control. Subsequently, 100 µL of cell lysates was incubated with magnetic bead-IgG or Ago2 antibody complex at 4 °C overnight. The next day, the complex was washed according to the manufacturer's instructions. RNA was then extracted and purified. The level of hsa_circ_0081343 in the purified RNA was detected by qRT-PCR.

### Statistical analysis

Statistical analyses were performed using SPSS 18.0 and GraphPad Prism version 7.0 software. Data are expressed as means ± standard deviations based on three independent experiments. Differences between two groups were analyzed using unpaired *t*-tests when the data were normally distributed or non-parametric *t*-tests when the data were not normally distributed. Differences between more than two groups were analyzed using a one-way analysis of variance. *P* values < 0.05 were considered statistically significant.

## Results

### Hsa_circ_0081343 expression is significantly lower in FGR placenta as compared to healthy placenta

**Fig. 1:**
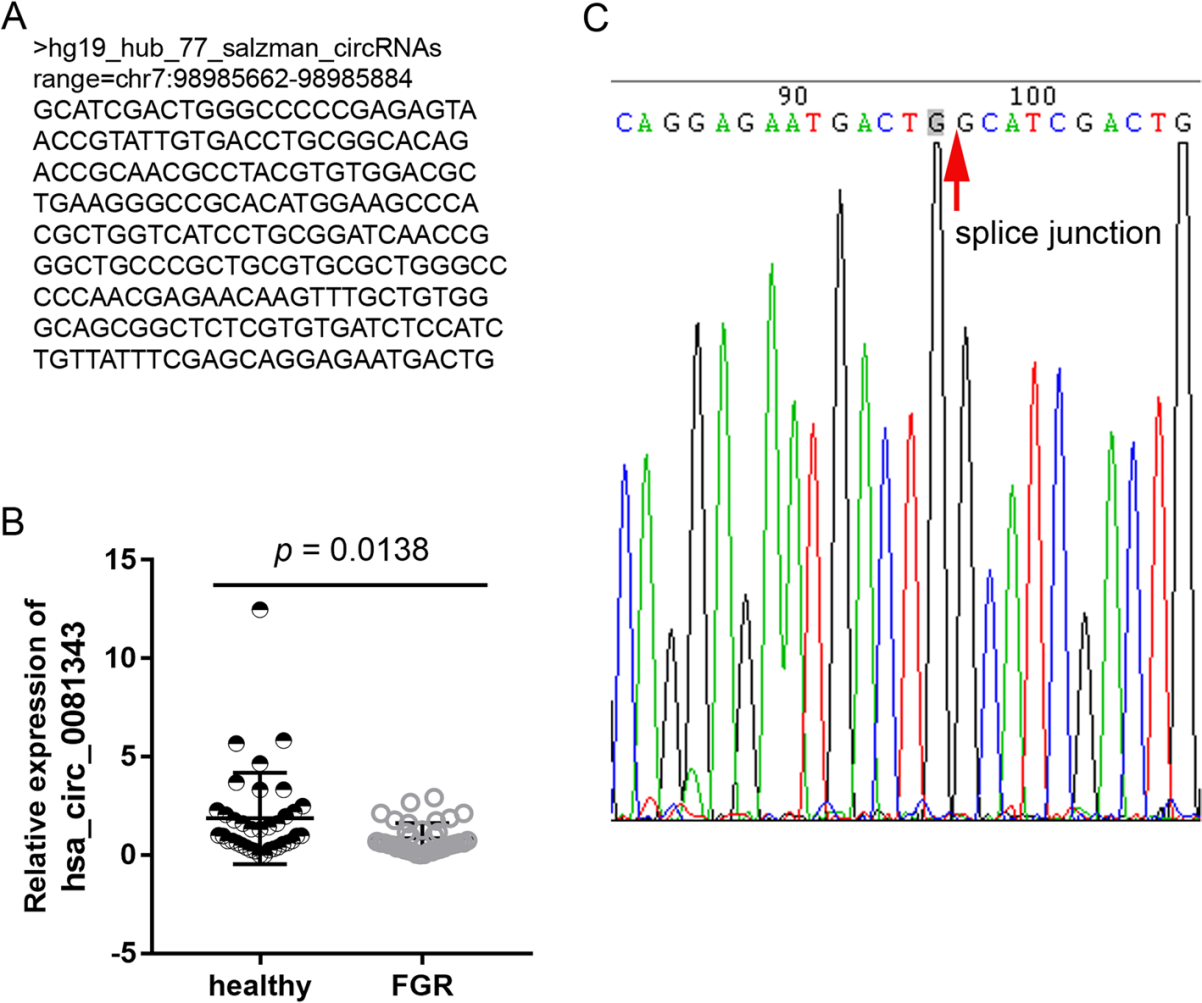
Hsa_circ_0081343 expression is downregulated in FGR placenta compared to expression in healthy controls. **A**: hsa_circ_0081343 sequence reported in circBase database. **B**: hsa_circ_0081343 expression level in FGR and healthy placental tissues (n = 37). **C**: the splice junction of hsa_circ_0081343 shown in circBase verified by sequencing the DNA fragment from qRT-PCR

In our previous study [12], the circRNA microarray results showed that hsa_circ_0081343 expression levels were lower in FGR placental tissues than in healthy placental tissues. The sequences reported in the circBase database are shown in Fig. 1A. In the present study, we further investigated the expression profile in 37 pairs of clinical samples. The results of qRT-PCR showed that hsa_circ_0081343 expression levels were significantly downregulated in the 37 FGR placental tissues as compared to the 37 healthy placental tissues (Fig. 1B). Sequencing analysis of the DNA fragment by qRT-PCR verified the presence of the splice junction of hsa_circ_0081343 as shown in circBase (Fig. 1C).

### Hsa_circ_0081343 promotes migration and invasion and inhibits apoptosis in HTR-8 cells

**Fig. 2:**
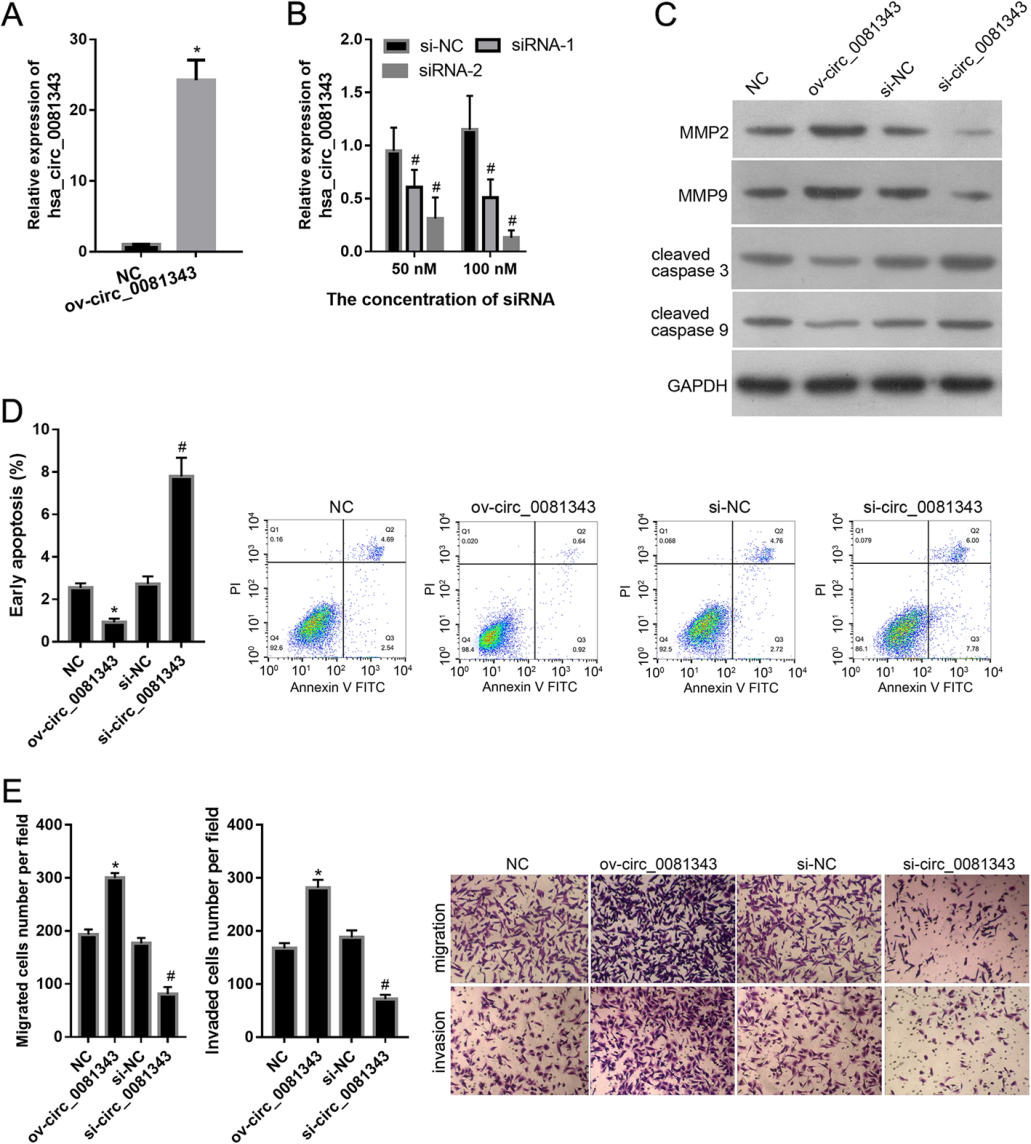
Effect of hsa_circ_0081343 overexpression or knockdown on the migration, invasion, and apoptosis of HTR-8 cells. **A**: hsa_circ_0081343 was overexpressed in HTR-8 cells by transfecting with hsa_circ_0081343 overexpression plasmid (ov-circ_0081343). Transfection with empty pLCD5H-ciR plasmid was used as a negative control (NC). B: hsa_circ_0081343 was silenced in HTR-8 cells by transfecting with two small interfering RNAs (siRNA-1 and -2) targeting hsa_circ_0081343. Transfection with negative control siRNA (si-NC) was used as control. **C**-**E**: The effect of hsa_circ_0081343 overexpression or silencing on migration- and apoptosis-related proteins (**C**), apoptosis (**D**), and the migration and invasion potential of HTR-8 cells (**E)**. *p < 0.05, when compared to NC; ^#^*p* < 0.05, when compared to si-NC. All experiments were independently performed three times

Considering the dysregulated expression of hsa_circ_0081343 in FGR placental tissues, we further explored its function by overexpressing or silencing hsa_circ_0081343 in HTR-8 cells. Our results showed that hsa_circ_0081343 expression levels were increased in HTR-8 cells upon transfection with ov-circ_0081343 as compared to the cells transfected with the empty vector or NC group (Fig. 2A). Furthermore, siRNA-2 demonstrated the highest efficacy in silencing the expression of hsa_circ_0081343 at 100 nM when compared to that of the si-NC group (Fig. 2B). In subsequent assays, the transfection of siRNA-2 at 100 nM was termed the si-circ_0081343 group. Overall, these results demonstrate that hsa_circ_0081343 was successfully overexpressed or silenced in HTR-8 cells.

Subsequently, we examined the effect of hsa_circ_0081343 overexpression or knockdown on the migration, invasion, and apoptosis of HTR-8 cells. Our immunoblotting studies revealed that both MMP2 and MMP9 levels increased upon circ_0081343 overexpression in HTR-8 cells (ov-circ_0081343). However, their levels were downregulated when circ_0081343 expression was silenced (si-circ_0081343) as compared to the negative control siRNA (si-NC) (Fig. 2C). Furthermore, cleaved caspase 3 and cleaved caspase 9 levels were found to be lower in the ov-circ_0081343 group than in the NC group, but were higher in the si-circ_0081343 group than in the si-NC group (Fig. 2C). Flow cytometry analysis revealed that the percentage of early apoptotic cells was lower in the ov-circ_0081343 group than in the NC group, but was higher in the si-circ_0081343 group than in the si-NC group (Fig. 2D). The results of the Transwell assay further revealed that the number of migrated or invaded cells per field was higher in the ov-circ_0081343 group than in the NC group, but was lower in the si-circ_0081343 group than in the si-NC group (Fig. 2E). Overall, these results indicate that hsa_circ_0081343 overexpression promotes cell migration and invasion and inhibits apoptosis in HTR-8 cells. However, silencing hsa_circ_0081343 produces the opposite effects.

### Hsa_circ_0081343 interacts with miR-210-5p

**Fig. 3:**
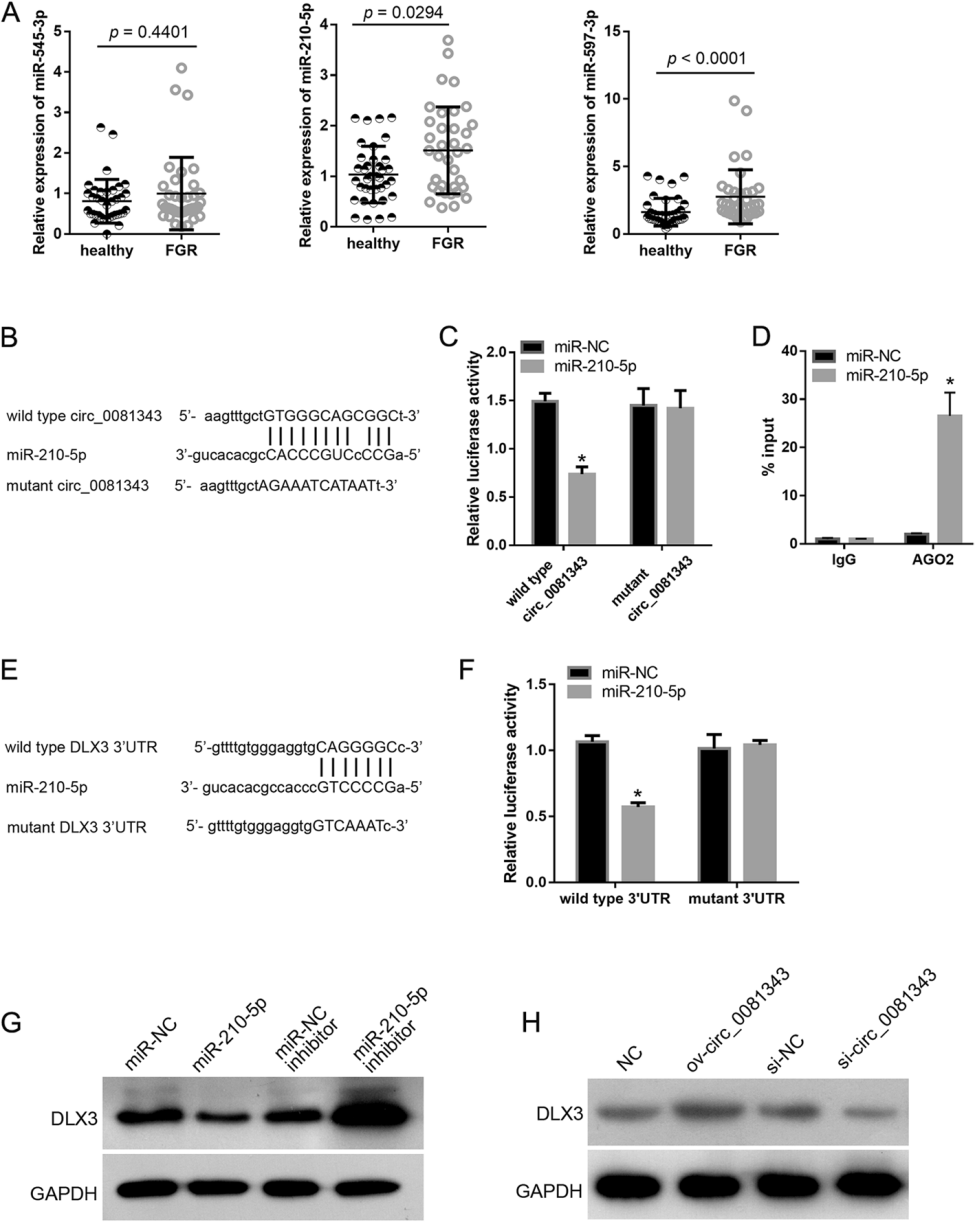
Hsa_circ_0081343 physically interacts with miR-210-5p and miR-210-5p directly targets DLX3 transcript. **A**: Expression levels of miR-545-3p, miR-210-5p, and miR-597-3p in FGR and healthy placental tissues (n = 37). **B**: The binding site of miR-210-5p on hsa_circ_0081343 sequence, and the mutant scheme of luciferase reporter vector containing linear hsa_circ_0081343 with mutated miR-210-5p binding site. **C**: miR-210-5p mimic co-transfection decreases the relative luciferase activity of luciferase reporter vector containing wild type linear hsa_circ_0081343 (wild type circ_0081343). **D**: miR-210-5p mimic transfection increases hsa_circ_0081343 level in RNAs enriched by AGO2. **E**: The binding site of miR-210-5p on 3′ UTR of DLX3, and the mutant scheme of luciferase reporter vector containing 3′ UTR of DLX3 with mutated miR-210-5p binding site. **F**: miR-210-5p mimic co-transfection decreases the relative luciferase activity of luciferase reporter vector containing wild type 3′ UTR of DLX3 (wild type 3′ UTR). **G**-**H**: Effect of the overexpression or knockdown of miR-210-5p or hsa_circ_0081343 on DLX3 protein level. *p < 0.05, when compared to miR-NC. The experiments in panels **A**, **C**, **D**, **F**, **G**, and **H** were independently performed three times

It has been reported that circRNAs function as ceRNAs. Thus, we analyzed the presence of miRNA response elements in the hsa_circ_0081343 sequence. Based on the sequence complementary matching score of hsa_circ_0081343 and miRNAs, the top three miRNA candidates, namely miR-545-3p, miR-210-5p, and miR-597-3p, were chosen for further studies. The results of qRT-PCR showed that miR-210-5p and miR-597-3p expression levels were significantly upregulated in the 37 FGR placental tissues compared to the levels in the 37 healthy placental tissues (Fig. 3A). A review of the literature revealed that miR-210-5p is a hypoxia-regulated miRNA [13]. Moreover, hypoxia is frequently associated with FGR [14]. Consequently, we focused on the relationship between miR-210-5p and hsa_circ_0081343. The binding site of miR-210-5p on hsa_circ_0081343 is shown in Fig. 3B. Additionally, we identified their relationship using a dual luciferase activity assay and an anti-AGO2 RIP assay. The results of the dual luciferase activity assay revealed that the relative luciferase activity of the miR-210-5p mimic plus wild type circ_0081343 co-transfection group was evidently lower than that of the miR-NC plus wild-type circ_0081343 co-transfection group; however, the relative luciferase activity was not different between the miR-210-5p mimic plus mutant circ_0081343 co-transfection group and the miR-NC plus mutant circ_0081343 co-transfection group (Fig. 3C). These results suggest that miR-210-5p can bind to the linear sequence of hsa_circ_0081343. The results of the anti-AGO2 RIP assay indicated that miR-210-5p mimic transfection increased the hsa_circ_0081343 level in RNAs enriched with AGO2 (Fig. 3D), indicating that miR-210-5p interacts with hsa_circ_0081343 in HTR-8 cells.

### DLX3 is a direct target of miR-210-5p

To explore the mechanism underlying the hsa_circ_0081343/miR-210-5p axis, we predicted the targets of miR-210-5p. Among all predicted targets, DLX3 is dysregulated in idiopathic FGR placentae [15]. Thus, we explored whether DLX3 is a target of miR-210-5p and whether the hsa_circ_0081343/miR-210-5p axis regulates DLX3. The binding site of miR-210-5p on the 3′ UTR of DLX3 is shown in Fig. 3E. The results of the dual luciferase activity assay showed that the relative luciferase activity of the miR-210-5p mimic + wild type 3′ UTR co-transfection group was evidently lower than that of the miR-NC + wild type 3′ UTR co-transfection group. However, the relative luciferase activity remained unchanged between the miR-210-5p mimic + mutant 3′ UTR co-transfection group and miR-NC + mutant 3′ UTR co-transfection group (Fig. 3F). These results suggest that miR-210-5p can directly interact with the 3′ UTR of DLX3. The results of western blotting showed that DLX3 protein levels were downregulated in HTR-8 cells upon overexpression of miR-210-5p compared to the levels in the miR-NC group (Fig. 3G). Additionally, DLX3 levels were found to be increased in HTR-8 cells upon transfection with the miR-210-5p inhibitor compared to the levels in the miR-NC inhibitor group (Fig. 3G). Furthermore, the DLX3 protein levels were found to be higher in the ov-circ_0081343 group than in the NC group, but lower in the si-circ_0081343 group than in the si-NC group (Fig. 3H).

### miR-210-5p overexpression rescues the effect of hsa_circ_0081343 overexpression in HTR-8 cells

**Fig. 4:**
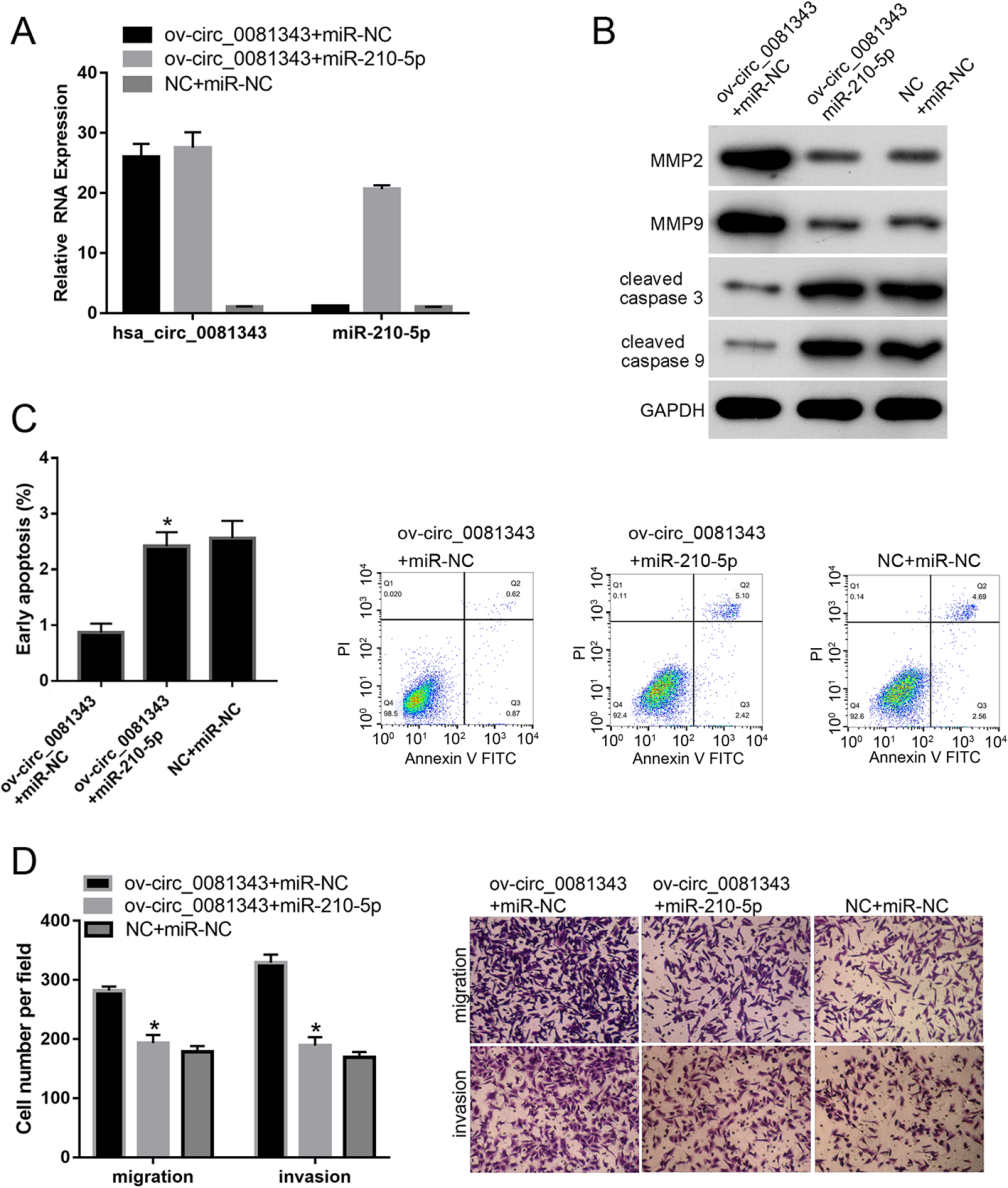
miR-210-5p overexpression alleviates the effect of hsa_circ_0081343 overexpression in HTR-8 cells. HTR-8 cells were transfected with ov-circ_0081343 + miR-NC, ov-circ_0081343 + miR-210-5p, or NC + miR-NC. The overexpression of miR-210-5p and hsa_circ_0081343 in the three groups was verified by qRT-PCR. The protein levels of MMP2, MMP9, cleaved caspase 3, and cleaved caspase 9 (**B**), early apoptosis level (**C**), and the migration and invasion potential (**D**) of HTR-8 cells under the indicated co-transfection conditions were evaluated by western blotting, flow cytometry analysis, and Transwell assays, respectively. *p < 0.05, when compared to ov-circ_0081343 + miR-NC group. All experiments were independently performed three times

To further confirm whether hsa_circ_0081343 acts as a ceRNA for miR-210-5p, we evaluated whether miR-210-5p overexpression could rescue the effect of hsa_circ_0081343 overexpression in HTR-8 cells. Our qRT-PCR results revealed that the expression of hsa_circ_0081343 was higher in the ov-circ_0081343 + miR-NC and ov-circ_0081343 + miR-210-5p groups than in the NC + miR-NC group (Fig. 4A). In addition, the expression of miR-210-5p was higher in the ov-circ_0081343 + miR-210-5p groups than in both the ov-circ_0081343 + miR-NC and NC + miR-NC groups (Fig. 4A). These results suggest that miR-210-5p and hsa_circ_0081343 were successfully overexpressed in the HTR-8 cells. The results of western blotting showed that the expression of MMP2 and MMP9 was decreased in the ov-circ_0081343 + miR-210-5p group compared to that in the ov-circ_0081343 + miR-NC group (Fig. 4B). Cleaved caspase 3 and cleaved caspase 9 levels were, in contrast, found to be higher in the ov-circ_0081343 + miR-210-5p group than in the ov-circ_0081343 + miR-NC group (Fig. 4B). Our flow cytometry data revealed that the percentage of early apoptotic cells was higher in the ov-circ_0081343 + miR-210-5p group than in the ov-circ_0081343 + miR-NC group (Fig. 4C). The results of the Transwell assay revealed that the number of migrated or invaded cells per field was lower in the ov-circ_0081343 + miR-210-5p group than in the ov-circ_0081343 + miR-NC group (Fig. 4D). These results indicate that miR-210-5p overexpression alleviates the effect of hsa_circ_0081343 overexpression on the migration and invasion potential, as well as the apoptosis of HTR-8 cells.

### Inhibition of miR-210-5p suppresses apoptosis and promotes migration and invasion of HTR-8 cells

**Fig. 5:**
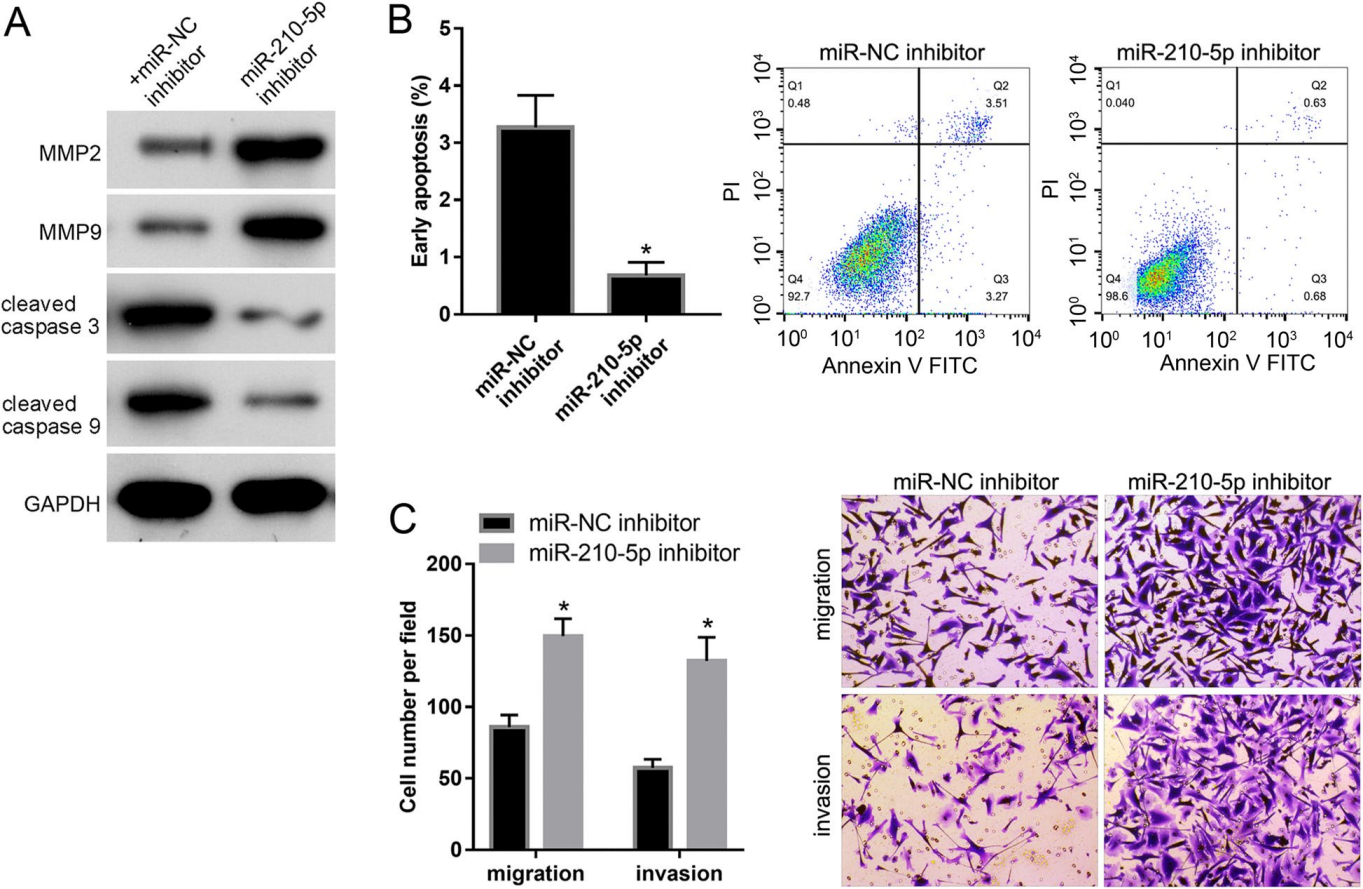
miR-210-5p inhibitor suppresses apoptosis and stimulates migration and invasion of HTR-8 cells. HTR-8 cells were transfected with miR-NC, ov-circ_0081343 + miR-210-5p, and NC + miR-NC. The overexpression of miR-210-5p and hsa_circ_0081343 in three groups was verified by qRT-PCR. The protein levels of MMP2, MMP9, cleaved caspase 3, and cleaved caspase 9 (**B**), early apoptosis level (**C**), and the migration and invasion capacities (**D**) were evaluated by western blot, flow cytometry analysis, and Transwell assays, respectively. *p < 0.05, when compared to ov-circ_0081343 + miR-NC group; * *p* < 0.05, when compared to miR-NC inhibitor. All experiments were independently performed three times

To evaluate the potential role of miR-210-5p in FGR, we analyzed the effect of miR-NC and miR-210-5p inhibitors on apoptosis, migration, and invasion of HTR-8 cells. The results of western blotting studies showed that both MMP2 and MMP9 expression levels were upregulated in the miR-210-5p inhibitor groups when compared to the miR-NC inhibitor group (Fig. 5A). In contrast, the cleaved caspase 3 and cleaved caspase 9 levels were downregulated in the miR-210-5p inhibitor group when compared to the miR-NC inhibitor group (Fig. 5A). Flow cytometry analysis indicated that the percentage of cells undergoing early apoptosis was lower in the miR-210-5p inhibitor group than in the miR-NC inhibitor group (Fig. 5B). The results of the Transwell assay revealed that the migration or invasion of cells per field was evidently higher in the miR-210-5p inhibitor group than in the miR-NC inhibitor group (Fig. 5C). Overall, these results suggest that the inhibition of miR-210-5p suppresses apoptosis and promotes the migration and invasion of HTR-8 cells.

## Discussion

In the present study, we found that the expression of hsa_circ_0081343 was significantly downregulated in FGR placental tissues compared to that in healthy controls. Sequence analysis of the qRT-PCR DNA fragment verified that hsa_circ_0081343 is a genuine circRNA. Hence, we analyzed its role in HTR-8 cells. We found that hsa_circ_0081343 overexpression decreased, while its knockdown increased the apoptosis level of HTR-8 cells. Moreover, we found that hsa_circ_0081343 overexpression could enhance and hsa_circ_0081343 knockdown could weaken the migration and invasion capabilities of HTR-8 cells. HTR-8 is a cell line derived from human extravillous trophoblasts. In the human placenta, there are three major trophoblast subpopulations: cytotrophoblasts, extravillous cytotrophoblasts, and syncytiotrophoblasts. Hence, our results suggest that circ_0081343 plays a role in regulating placental trophoblast cell function. To the best of our knowledge, our study is the first to analyze the functional role of hsa_circ_0081343. Previous studies have shown that the apoptosis level of placental trophoblasts is lower in healthy pregnancies than in FGR, and this may contribute to the placental pathology of FGR [16, 17]. Extravillous trophoblast cells migrate to and invade the uterine wall, leading to remodeling of the maternal vasculature [18–20]. The maintenance of migration and invasion capabilities of extravillous trophoblast cells is important for the normal development of the fetus [21]. Moreover, HTR-8 cells are usually used as a cell model of FGR. Therefore, we speculated that hsa_circ_0081343 may play a regulatory role in the pathogenesis of FGR. However, further experiments are required to support this speculation. In future studies, we will investigate the role of hsa_circ_0081343 in animal models of FGR.

CircRNAs bind to microRNAs and function as “miRNA sponges.” As a result, the suppression effect of miRNAs on target genes is relieved, resulting in increased target gene expression. This regulatory mechanism is termed the competitive endogenous RNA (ceRNA) mechanism [22, 23]. To explore the molecular mechanism of hsa_circ_0081343 from the perspective of ceRNA, we investigated the relationship between hsa_circ_0081343 and miR-210-5p. Our results demonstrated that hsa_circ_0081343 can directly bind to miR-210-5p in HTR-8 cells. Our results showed that miR-210-5p overexpression relieved the effect of hsa_circ_0081343 overexpression in HTR-8 cells. These results suggest that hsa_circ_0081343 functions as a ceRNA by sponging miR-210-5p. This hypothesis is further supported by the promotion of apoptosis and suppression of the migration and invasion capabilities of HTR-8 cells upon transfection with miR-210-5p inhibitor, as shown in Fig. 5.

According to the ceRNA mechanism, the circRNA/miRNA axis plays a crucial role in biological phenomena by affecting the translation of target mRNAs. Therefore, it is vital to identify the target of hsa_circ_0081343/miR-210-5p to fully elucidate the molecular mechanisms involved. DLX3 is a member of the homeodomain transcription factor and vertebrate-free distant homeobox gene family. Its function and regulatory mechanisms in the placenta have been previously reported [24, 25]. DLX3 is required for the generation of a functional chorioallantoic placenta, and targeted deletion of the mouse DLX3 gene results in embryonic death between days 9.5 and 10 due to placental defects [26]. Chui et al. reported that DLX3 is expressed in proliferating and differentiating cells of the human placenta; thus, DLX3 may play an important role in normal placental development [24]. Our present study demonstrated that miR-210-5p can bind to the 3′ UTR of DLX3 and decrease the protein level of DLX3. These results indicate that DLX3 is a direct target of miR-210-5p. Moreover, hsa_circ_0081343 overexpression increased the protein levels of DLX3. Therefore, we hypothesized that DLX3 is the target of the hsa_circ_0081343/miR-210-5p axis and that hsa_circ_0081343 promotes trophoblast cell migration and invasion and inhibits trophoblast apoptosis by regulating the miR-210-5p/DLX3 axis.

The role of hsa_circ_0081343 in HTR-8 cells is similar to that of hsa_circ_0000848, which was reported in our previous study [12]. Therefore, we predicted that there may be crosstalk between hsa_circ_0081343 and hsa_circ_0000848. hsa_circ_0000848 promotes migration and invasion and inhibits apoptosis by sponging hsa-miR-6768-5p [12]. However, we did not identify the target of hsa-miR-6768-5p in our previous study. In the present study, we identified DLX3 as a target of the hsa_circ_0081343/miR-210-5p axis. Identification of the target gene can further explain the molecular mechanisms of hsa_circ_0081343. By predicting the target of hsa-miR-6768-5p using TargetScanHuman 7.1 online software, we found that DLX3 is also a potential target of hsa-miR-6768-5p (data not shown). Hence, we hypothesized that hsa_circ_0081343 and hsa_circ_0000848 play similar roles by affecting DLX3 levels.

While our study conclusively shows that hsa_circ_0081343 regulates the cellular function of HTR-8 cells, it also has certain limitations. First, numerous miRNA response elements were found to be present in the hsa_circ_0081343 sequence. Hence, the existence of other miRNAs as potential downstream targets cannot be ruled out. In addition, the function of hsa_circ_0081343 in HTR-8 cells does not conclusively demonstrate that hsa_circ_0081343 regulates the pathogenesis of FGR. In future experiments, it will be necessary to construct an FGR animal model to study the effects of hsa_circ_0081343 in vivo.

In conclusion, our study demonstrates that hsa_circ_0081343 expression levels are significantly downregulated in FGR placental tissues. In vitro assays have shown that hsa_circ_0081343 promotes migration and invasion and inhibits apoptosis via the hsa-miR-210-5p/DLX3 axis in HTR-8 cells. Our results suggest that circ_0081343 plays a role in regulating placental trophoblast cell function.

## Data Availability

The data from this study are available in this published article.
